# When two actors perform different tasks: Still no evidence for shared task-sets in joint task switching

**DOI:** 10.1177/17470218211031545

**Published:** 2021-07-09

**Authors:** Motonori Yamaguchi, Husnain H Shah, Bernhard Hommel

**Affiliations:** 1 Department of Psychology, University of Essex, Colchester, UK; 2 Department of Psychology, Edge Hill University, Ormskirk, UK; 3 Institute of Psychology, Leiden University, Leiden, The Netherlands

**Keywords:** Task sharing, joint task switching, co-representation, joint cognition

## Abstract

Two different variations of joint task switching led to different conclusions as to whether co-acting individuals share the same task-sets. The present study aimed at bridging this gap by replicating the version in which two actors performed two different tasks. Experiment 1 showed switch costs across two actors in a joint condition, which agreed with previous studies, but also yielded even larger switch costs in a solo condition, which contradicted the claim that actors represent an alternative task as their own when it is carried out by the co-actor but not when no one carries it out. Experiments 2 and 3 further examined switch costs in the solo condition with the aim to rule out possible influences of task instructions for and experiences with the other task that was not assigned to the actor. Before participants were instructed on the second of the two tasks, switch costs were still obtained without a co-actor when explicit task names (“COLOUR” and “SHAPE”) served as go/nogo signals (Experiment 2), but not when arbitrary symbols (“XXXX” and “++++”) served as go/nogo signals (Experiment 3). The results thus imply that switch costs depend on participants’ knowledge of task cues being assigned to two different tasks, but not on whether the other task is performed by a co-actor. These findings undermine the assumption that switch costs in the joint conditions reflect shared task-sets between co-actors in this procedure.

Numerous models and theories have been suggested to account for human performance in isolation but only little is known about how individuals perceive and act in the presence of, or in interaction with, other individuals (Knoblich & Sebanz, 2006). This lack of knowledge has, among other things, motivated studies in which individual performance in classical experimental tasks is compared with performance in conditions where the task is shared with a co-actor. The studied tasks comprised the Simon task (e.g., Sebanz et al., 2003; Yamaguchi et al., 2018), flanker tasks (e.g., Atmaca et al., 2011; Dolk, Hommel, Prinz, & Liepelt, 2014), and task-switching designs (Dudarev & Hassin, 2016; Liefooghe, 2016; Yamaguchi et al., 2017b), on which our present study focused—the key question being how much an actor represents of his or her co-actor’s task (Wenke et al., 2011; Yamaguchi et al., 2017b).

When performed in isolation, a typical task switching procedure consists of two different tasks (e.g., Mayr & Kliegl, 2000), and a robust finding is that responses are faster when the current trial requires the same task as the immediately preceding trial (*task repeat trial*) than when it requires switching to a different task (*task switch trial*). The difference in response time (RT) between these types of trials is called *task-switch costs* (TSCs), or simply *switch costs*. Although robust, TSCs can be abolished when the preceding trial is a nogo trial, which is mainly because the advantage of repeating the same task disappears when a response is withheld on the preceding trial (Schuch & Koch, 2003).

Our previous studies used a joint version of this go/nogo procedure with pairs of actors sharing the tasks (Yamaguchi et al., 2017b): each actor was assigned the same set of two tasks, but each trial required only one of the actors to make a response (a go trial) whereas the other actor had to withhold responding (a nogo trial). The results showed TSCs when the preceding trial was performed by the same actor as the actor on the current trial (i.e., after a go trial), but they were abolished when the preceding trial was performed by a different actor than the current actor (after a nogo trial). The outcomes were comparable to those in a *solo* condition in which the actors performed a go/nogo procedure without a co-actor (Schuch & Koch, 2003). These results were replicated in subsequent studies (Yamaguchi et al., 2017a, 2019) and suggest that actors do not represent the task of their co-actor.

The exact opposite conclusion was drawn by Dudarev and Hassin (2016), who had participants switch between a parity and a magnitude task. In a full-task condition, participants were presented with a task cue that indicated which task they were to perform and, 900 ms later, with a stimulus that required a left–right keypress judgement regarding its parity or magnitude. In a solo condition, they carried out only one of the two tasks but did nothing on trials where the task cue indicated the other task. In a joint condition, they did the same but here the other task was carried out by a co-actor. The important feature of Dudarev and Hassin’s procedure was that each actor was assigned a single task that differed from their co-actor’s task, whereas each actor was assigned the same set of two tasks in our previous joint task switching (Yamaguchi et al., 2017b). In Dudarev and Hassin’s procedure, TSCs were obtained in the full-task condition and in the joint condition, but not in the solo condition. Note that this finding of TSCs in the solo condition appears inconsistent with findings in many previous studies, in which TSCs were abolished after nogo trials (e.g., Lenartowicz et al., 2011; Schuch & Koch, 2003; Verbruggen et al., 2005), but Dudarev and Hassin’s procedure differed from those used in the previous studies: given that each participant performed only one of the two tasks, trials following nogo trials were always switch trials.

Dudarev and Hassin’s second experiment generated a “task-switching benefit” in the solo condition and a TSC in the joint condition. Importantly, in their third condition in which two actors carried out the same task, they found no effect, based on which the authors concluded that the effect found in their joint condition was not due to switching between actors. Instead, the authors concluded that “people track others’ tasks and mentally do it with them, even when doing it engages effortful and costly executive functions” (p. 227). In other words, the actors *co-represented* their co-actor’s task and performed it mentally as if it were their own task (Knoblich & Sebanz, 2006). If their conjecture is correct, Dudarev and Hassin’s procedure could be a viable method to investigate task sharing between co-actors.

Interestingly, Liefooghe (2016) conducted a study very similar to Dudarev and Hassin’s (2016) study but with somewhat different outcomes. He had participants switch between colour and shape judgements of visual stimuli, as indicated by a task cue that preceded the stimuli by either 100 or 1,000 ms. He also compared a full-task condition with solo and joint conditions, both of which required each actor to perform only one of the two tasks. The full-task condition again generated the largest TSC, which was about three times larger than the TSC in the joint condition. With the long cue–stimulus interval, the joint condition produced significant TSCs whereas the solo condition did not, which replicated Dudarev and Hassin (2016). With the short interval, however, significant TSCs were obtained in all three conditions, and TSCs in the joint and solo conditions were no longer different. Liefooghe (2016) concluded that participants in the joint condition “do not seem to make a representation of the co-actor’s task-set” (p. 72), and he considered that the remaining differences between the joint and solo conditions might be because the presence of the co-actor and/or of his or her activities impairs the retrieval of the actors own task rules and/or makes actor discrimination (which in these conditions is confounded with task discrimination) more difficult. Moreover, Liefooghe suggested that his use of peripheral left and right stimuli to spatially cue a task (and an actor) might have distracted participants in the nogo trials of the solo condition, where the cues actually drew attention away from the events belonging to the other task. Less peripheral cues, he speculated, might have created better comparability between the joint and solo conditions.

Taken altogether, the available evidence from Dudarev and Hassin (2016) and Liefooghe’s (2016) studies shows that TSCs can be obtained in the joint condition of their procedure, unlike the joint condition of our previous studies (Yamaguchi et al., 2017a, 2017b, 2019) and such a finding suggests that the presence of another person might indeed have an impact on task-switching performance of individuals. However, it remains unclear whether this impact really implies co-representation of tasks as suggested by Dudarev and Hassin, and whether and to what degree it challenges our previous conclusion that actors do not represent their co-actor’s task-set. Following Liefooghe, we considered the possibility that the critical difference between the joint and solo conditions, which led Dudarev and Hassin (2016) to argue for co-representation, might be due to differences in the attentional demands of these two conditions—differences that can account for the outcomes without referring to dedicated “social mechanisms.” We tested this possibility by partially replicating the basic experimental set-up of Dudarev and Hassin (2016) and Liefooghe (2016), except that we (a) focused on the theoretically relevant joint and solo conditions; (b) replaced Liefooghe’s potentially problematic peripheral task cues by central task cues; and (c) reduced display complexity as compared to Liefooghe by not presenting the stimulus–response mapping on the screen. According to Dudarev and Hassin’s (2016) account, one would expect TSCs to be restricted to the joint condition, whereas Liefooghe’s interpretation suggests that both conditions might generate TSCs.

## Experiment 1

The present experiment attempted to replicate the main findings of Dudarev and Hassin’s (2016) and Liefooghe’s (2016) versions of joint task switching in which two actors performed different tasks, with a central task cue and a simpler display than those used by Liefooghe. Each trial started with the task cue (“COLOUR” or “SHAPE) centrally presented on the display, which indicated one of the two tasks. In the joint condition, the actor to whom the cued task was assigned responded to the target on that trial while the other actor withheld responding. The solo condition was essentially identical with the joint condition except that only one actor responded when the assigned task was cued while no one responded when the co-actor’s task was cued. To obtain clear evidence for co-representation, we should obtain not only (a) significant TSCs in the joint condition but also (b) no TSCs in the solo condition, because the lack of significant TSCs in the solo condition would allow one to assert the importance of the co-actor for significant TSCs in the joint condition (Dudarev & Hassin, 2016).

### Method

#### Participants

Seventy-six undergraduate students at Edge Hill University participated in the present experiment as part of seminar in an introductory psychology module. One pair was discarded as one of the actors used wrong response keys throughout the joint task, which resulted in unusually low accuracy. Therefore, the final sample consisted of 74 participants (67 females; mean age = 18.95, *SD* = 2.93, range = 18–42). All participants reported having normal or corrected-to-normal visual acuity and normal colour vision, and they received experimental credits towards their module were paid £4 for participation. Participants were naïve as to the purpose of the experiment. All participants were provided with a participant information sheet at the beginning of the seminar, which described the nature of the task and the conditions of participation, and signed a consent form if they agreed to participate. The research protocol was approved by the Research Ethics Committee of the Psychology Department at Edge Hill University.

#### Apparatus and stimuli

The apparatus consisted of a 23-in flat screen monitor and a personal computer with a desktop QWERTY keyboard. The target stimuli were coloured shapes, green and red squares (4.8 cm in sides) and green and red diamonds (squares tilted 45°), and the task cues were the words “COLOUR” and “SHAPE” in 40-pt Arial font printed in black against a white background. Both stimuli appeared at the screen centre.

#### Procedure

The experiment was conducted in one afternoon. Participants were divided into four groups of 14–22 participants each. Two groups were run in parallel in two computer rooms that were located next to each other and had the same, but mirror-reversed, layout. Each room contained 24 seats that were arranged in four rows of six computers in each. At most three computers were used from each row, so that pairs of participants were seated every other computer to keep sufficient distances between pairs. Participants were randomly paired from different seminar groups by the experimenter and were instructed to sit in front of the computer monitors. Participants being placed on the left side were designated as “Actor A” and those on the right side as “Actor B” in the instructions that were presented on the computer monitor.

Participants read the instructions and started the task at their own pace by pressing the space bar. Participants were assigned to the colour task or the shape task in a random fashion. Actor A used the “z” and “c” keys as the left and right response keys, and Actor B the “1” and “3” keys on the numerical keypad. The assignment of response keys to the target values (green vs. red for the colour task, or square vs. diamond for the shape task) was also randomly determined by the computer at the beginning of the session. Participants were also instructed not to talk with their partners during the task. One of the experimenters stayed in each room throughout the session.

Each pair performed two phases of the experiment: solo task and joint task. In the solo-task phase, only one actor from the pair performed the task and the other actor sat quietly. After the first actor, the second actor performed the task alone in a similar manner. Each actor started with a block of 8 practice trials, followed by two blocks of 96 test trials. In the joint-task phase, two actors performed the task together. This phase also started with a block of 8 practice trials, followed by two blocks of 96 test trials. Half of the trials in each block were the colour task and the other half were the shape task, and they occurred in a random order.

Each trial started with the task cue that appeared in the screen centre for 750 ms. The target replaced the task cue and remained on the screen for 1,500 ms or until a response key was pressed. When the response was correct, the message “Good” replaced the target; when the response was incorrect, the message “Error” occurred at the same position; and when there was no response, the message “Faster!” occurred, except when no response was required (i.e., nogo trials) in which case the message was “Good.” If a response was made on nogo trials, the message was “Do not respond!” When a wrong actor pressed a key, the message was “Not your turn!” RT was the interval between target onset and pression of a response key. A session took less than 30 min.

Note that the sequence of the two tasks varied randomly. Accordingly, about half of the trials were *repeat trials* for which the task was the same as that on the preceding trial, so that the actor performing the trial was also the same as the one on the preceding trial. The other half were *switch trials* for which the task was different from that on the preceding trial, so that the actor performing the trial was also different from the one on the preceding trial. Therefore, task switching and actor switching were confounded in this procedure, like in Dudarev and Hassin (2016) and Liefooghe’s (2016) studies. At the same time, switch trials were always trials that followed a nogo trial, whereas repeat trials were always trials that followed a go trial, so that task/actor switching was also confounded by the type of preceding trial (go vs. nogo).

### Results and discussion

**Figure 1.**
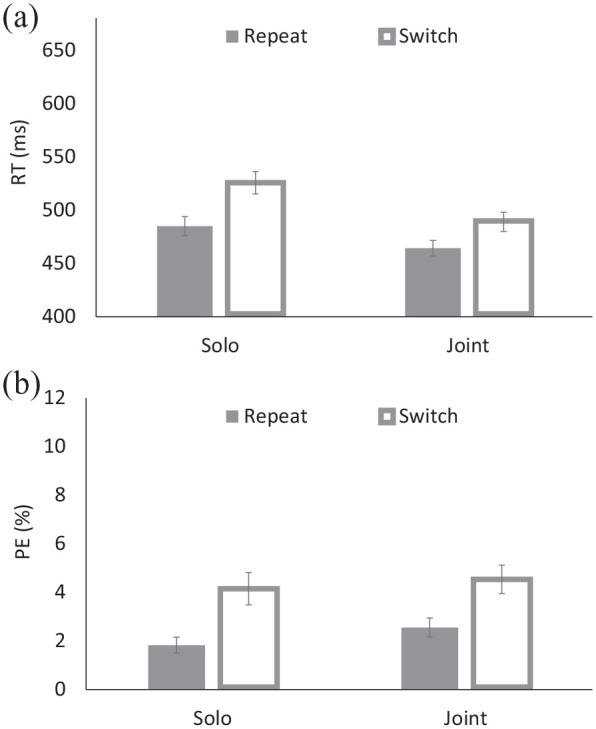
(a) Response times (RTs) and (b) percentages of errors (PEs) for the solo and joint tasks of Experiment 1.

Mean RT for correct go trials and percentages of error (PE) trials were computed for each actor. Trials were considered as an error if a wrong key was pressed or no response was made. Trials were discarded if RT was less than 200 ms, no response was made, or a wrong actor responded (1.52%). RT and PE were submitted to a 2 (Block: solo task vs. joint task) × 2 (Transition: repeat vs. switch) ANOVA (see Table 1), with both factors being within-subject variables. RT and PE are summarised in Figure 1.

**Table 1.** ANOVA results of Experiments 1–3.

Factors	*df*	*MSE*	*F*	*p*	* $\eta_{p}^{2}$ *
	Experiment 1: Response time				
**Block**	**1,73**	**2,384.95**	**33.16**	**<.001**	**.312**
**Transition**	**1,73**	**1,159.27**	**71.49**	**<.001**	**.495**
**Block × Transition**	**1,73**	**372.25**	**10.82**	**.002**	**.129**
	Experiment 1: Percentage of errors				
Block	1,73	11.87	<1	.963	<.001
**Transition**	**1,73**	**13.43**	**24.01**	**<.001**	**.248**
Block**×**Transition	1,73	10.19	1.01	.317	.014
	Experiment 2: Response time				
**Block**	**2,56**	**5,287.31**	**13.37**	**<.001**	**.323**
**Transition**	**2,56**	**2,720.17**	**52.02**	**<.001**	**.650**
Block**×**Transition	2,56	1,405.46	<1	.389	.033
	Experiment 2: Percentage of errors				
**Block**	**2,56**	**40.87**	**4.65**	**.014**	**.142**
**Transition**	**2,56**	**9.71**	**22.98**	**<.001**	**.451**
Block**×**Transition	2,56	6.87	1.40	.254	.048
	Experiment 3: Response time				
**Block**	**2,56**	**6,766.95**	**17.81**	**<.001**	**.389**
**Transition**	**2,56**	**1,977.41**	**41.86**	**<.001**	**.599**
**Block × Transition**	**2,56**	**1,949.31**	**6.87**	**.002**	**.197**
	Experiment 3: Percentage of errors				
**Block**	**2,56**	**51.56**	**3.85**	**.027**	**.121**
Transition	2,56	29.52	2.88	.101	.093
**Block × Transition**	**2,56**	**25.04**	**3.43**	**.039**	**.109**

Bold indicates statistical significance at α = .05.

MSE: mean squared error.

For RT, the main effects of Block and Transition were significant: responses were faster for the joint-task block (*M* = 434 ms) than for the solo-task block (*M* = 506 ms), and responses were faster for repeat trials (*M* = 473 ms) than for switch trials (*M* = 507 ms), yielding a 34-ms TSC. These factors interacted: TSCs were 41 ms for the solo-task block, and 26 ms for the joint-task block. Post hoc comparisons indicated that both TSCs were significant (*p* < .001).^1^ For PE, the only significant effect was a main effect of Transition: responses were more accurate for repeat trials (*M* = 2.26%) than for switch trials (*M* = 4.35%). The same Block-by-Transition interaction was obtained when using Dudarev and Hassin’s (2016) inverse efficiency scores, or IE = RT/(1–PE/100), *F*(1,73) = 7.87, *MSE* = 1035.35, *p* < .006, $\eta_{p}^{2}$ = .097—again, TSCs were numerically larger for the solo-task block (*M* = 56 ms) than for the joint-task block (*M* = 38 ms).

To summarise, our results replicated Dudarev and Hassin (2016) and Liefooghe’s (2016) findings of significant TSCs in the joint block. However, in contrast to these previous studies, we also found significant, and even larger TSCs in the solo-task block. The results suggest that the presence of an actively involved co-actor is not a necessary condition to obtain TSCs in the current task switching procedure. These outcomes provide little support for the idea of task or actor co-representation, because TSCs were not unique to the joint-task block. The conclusion corroborates those of our previous studies using different task switching procedures where two actors performed the same set of two tasks (Yamaguchi et al., 2017b), which also showed little differences between the joint and solo blocks.

## Experiment 2

Before further evaluating the outcome of Experiment 1 in light of our hypotheses, we aimed to test the possible role of some methodological differences between our first experiment and the two previous studies on joint task switching (Dudarev & Hassin, 2016; Liefooghe, 2016). Participants in the present Experiment 1 performed both the solo and joint conditions, whereas participants in the previous studies performed only one of these conditions. Accordingly, TSCs might have been obtained in the solo condition of Experiment 1 because at least some participants had already performed the joint-task block before the solo-task block, which might have introduced transfer effects, so that participants performed the solo-task block as if they were performing the joint-task block (see Ansorge & Wühr, 2009, for similar transfer effects between a choice-reaction task to a go/nogo task). While the analysis of order effects did not support this possibility (see Note 1), null-effects of ad hoc analyses do not provide the strongest evidence. It may be that merely being exposed to particular stimulus–response mappings during the instructions was sufficient to produce TSCs—perhaps because actors read the instructions for their co-actor as if they were instructions for their own (which could have led to instruction-based automaticity, see Liefooghe et al., 2012).

It should also be noted that participants in Experiment 1 performed the solo-task block in the presence of a co-actor who quietly sat on the next seat. Previous studies have shown that a mere presence of an inactive co-actor does not produce the joint Simon effect (e.g., Sebanz et al., 2003; Yamaguchi et al., 2018), but others also showed that the presence of a salient non-human object next to the participants could serve as a spatial reference and produce the joint Simon effect (Dolk, Hommel, Colzato, et al., 2014). Although a salient spatial reference is irrelevant to TSC, it is still possible that TSC in the solo condition were due to the presence of an inactive co-actor. This issue was also addressed in the present experiment.

In Experiment 2, participants performed the tasks alone. They were informed at the beginning of a session that there would be two different tasks, but they were not informed of the stimulus–response mappings for the second task before completing the first task. If instructing particular S-R mappings was responsible for TSCs in the solo condition, we should find TSCs in the second-performed task only, but not in the first-performed task.

### Method

#### Participants

A total of 29 participants (25 females, 4 males; mean age = 23.24, *SD* = 5.71, range = 18–42) were newly recruited from the Edge Hill University community. They received a £5 Amazon voucher for participation. All reported having normal or corrected-to-normal visual acuity and normal colour vision.

#### Apparatus, stimuli, and procedure

The apparatus was the same as that used in Experiment 1, but the experiment was conducted individually in a cubicle. Participants performed the solo go/nogo task of Experiment 1 in the first phase and the *full task* in the second phase, where they performed both of the two tasks and responded on every trial. In the solo go/nogo phase, participants were assigned either the colour or shape task and responded on trials only when the task cue indicated the assigned task and withheld responding when the other task was cued. There was one block of 8 practice trials, followed by two blocks of 96 test trials, as in Experiment 1. Then, the same participants switched the tasks, so that they responded on trials only when the task cue indicated the previously ignored task and withheld responding when the previously assigned task was cued. Again, there was a block of 8 practice trials, followed by 96 test trials. In the full-task phase, participants responded on all trials. It started with a block of 8 practice trials, followed by two test blocks of 96 trials.

Participants used the “z” and “c” keys for one task and the “1” and “3” keys on the numeric pad for the other task. In the solo go/nogo phase, they used the left and right index fingers of one hand to press the two keys (“z” and “c,” or “1” and “3”). In the full-task phase, they used their index and middle fingers of the left hand to press “z” and “c,” and the index and middle fingers of the right hand to press “1” and “3.” The assignments of the keys to the two shapes and colours, and to the two tasks, were randomly determined for each participant.

Participants were informed of the specific stimulus–response mappings for a given task only before they performed a block of trials for that task. Thus, those who started with the colour task did not know the stimulus–response mappings for the shape task until they completed the colour task of the solo go/nogo phase, and those who started with the shape task did not know the stimulus-response mappings for the colour task until they completed the shape task in the solo go/nogo phase. The procedure was as in Experiment 1 in all other respects.

### Results and discussion

**Figure 2.**
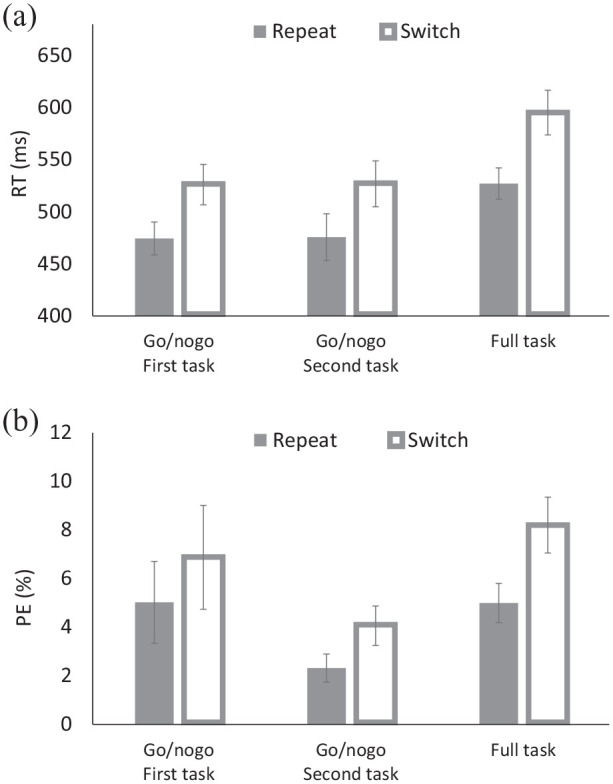
(a) Response times (RTs) and (b) percentages of errors (PEs) for the solo go/nogo task and the full task of Experiment 2.

Trials were filtered and mean RT and PE were computed in the same manner as in Experiment 1 (4.36% were discarded), and submitted to 3 (Block: first go/nogo vs. second go/nogo vs. full task) × 2 (Transition: repeat vs. switch) ANOVAs, with both factors being within-subject variables (see Table 1, Figure 2).

For RT, there were main effects of Block and of Transition. Responses were faster for the first and second go/nogo blocks (*M*s = 500 ms and 501 ms, respectively) than for the full-task block (*M* = 561 ms). Responses were faster for repeat trials (*M* = 492 ms) than for switch trials (*M* = 549 ms), yielding TSCs. However, these variables did not interact, indicating that TSCs were comparable for the three blocks. Importantly, TSCs were 52 ms for the first and 51 ms for the second go/nogo blocks.

For PE, there were also main effects of Block and Transition. Responses were most accurate for the second go/nogo task (*M* = 3.19%), intermediate for the first go/nogo block (*M* = 5.95%), and least accurate for the full-task block (*M* = 6.60%). Responses were also more accurate for repeat trials (*M* = 4.11%) than for switch trials (*M* = 6.38%). The interaction was not significant either. TSCs were again comparable for the first and second go/nogo blocks (*Ms* = 1.85% and 1.74% for the first and second blocks, respectively).

Both RT and PE indicated that TSCs are robust in the solo go/nogo conditions, and their comparable size in the two blocks suggests that the costs do not depend on whether other stimulus-response mappings have been presented to the actors before or whether an inactive co-actor was present next to the active actor.

## Experiment 3

Although participants were only instructed on stimulus–response mappings for one task in the first block of trials, the task cues were “COLOUR” and “SHAPE” which explicitly denoted the two tasks to be performed. This might have allowed participants to infer the second task to come in the later phase of the experiment without being informed of it explicitly. If so, TSCs obtained in the first block of Experiment 2 could be due to the anticipation of the two different tasks implied by the task cues. To rule out this possibility in Experiment 3, we replaced the task cues with arbitrary symbols (“XXXX” and “++++”) and assigned them randomly to the colour and shape tasks for each participant. We expected that TSCs would be obtained with the arbitrary task cues if merely switching between task cues produced the effect (Logan & Bundesen, 2003); however, if participants’ knowledge or anticipation of two tasks were responsible for TSCs in Experiment 2, there should be no TSCs in the first go/nogo block of the present experiment where participants had not been informed of the existence of the second task, but TSCs would emerge in the second go/nogo block where participants had been instructed on the second task.

### Method

#### Participants

Although the present experiment was planned as a laboratory experiment, we moved it to online data collection due to the ongoing global pandemic outbreak that started early in 2020. Thirty six participants from the University of Essex community completed the experiment, but seven were excluded due to low accuracy or a high portion of missing trials (see the “Results” section). Therefore, the present experiment included 29 participants (24 females, 5 females; mean age = 20.47, *SD* = 3.61, range = 18–38) who received experimental credits towards their psychology modules. All reported having normal or corrected-to-normal visual acuity and normal colour vision. The protocol was approved by the Research Ethics Committee at the University of Essex.

#### Apparatus, stimuli, and procedure

The experiment was created using lab.js (https://lab.js.org/), which was embedded within a Qualtrics survey (https://www.qualtrics.com/). Participants were not allowed to use tablet PCs or smartphones, as filtered by the survey function. The experiment ran on a browser, and it required either Google Chrome or Mozilla Firefox. To keep the display size as consistent across participants as possible, the experiment started with a calibration screen in which participants were asked to adjust a rectangle displayed on their computer monitor to the size of their credit card. The experimental programme can be viewed via the following link (https://sleepy-noether-418ecc.netlify.app).

The experiment followed Experiment 2 closely, with the following changes. First, the task cues were replaced with arbitrary strings “++++” and “XXXX,” instead of “COLOUR” and “SHAPE” used in Experiment 2. For half of the participants, ++++ was a go signal that required participants to respond to the target, and XXXX was a nogo signal that required participants to refrain from responding to the target in the first block of trials; XXXX was a go signal and ++++ was a nogo signal in the second block. For the other half, the meanings of the two cues were reversed. Second, responses were now made by pressing “a” and “d” for one task and “j” and “l” for the other task. This change was necessary because participants might not have used a keyboard with a numerical pad.

### Results and discussion

**Figure 3.**
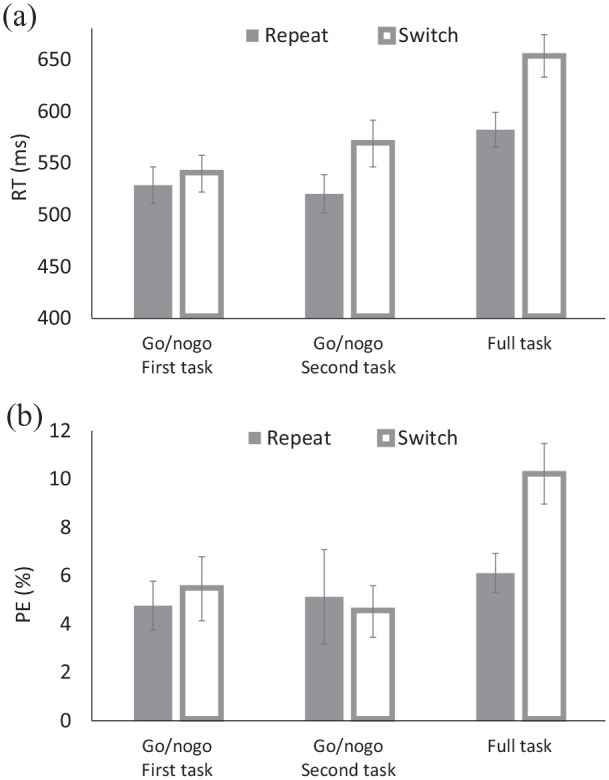
(a) Response times (RTs) and (b) percentages of errors (PEs) for the solo go/nogo task and the full task of Experiment 3.

From the 36 participants who completed the entire session, 4 participants were excluded for the overall response accuracy lower than 70%, and 3 participants were excluded for a high proportion of no response on go trials in the first two blocks (>45%). For the remaining 29 participants, trials were filtered in the same manner as in Experiment 2 (5.11% of the trials were discarded). Mean RT and PE were submitted to 3 (Block: first go/nogo vs. second go/nogo vs. full task) × 2 (Transition: repeat vs. switch) ANOVAs, with both factors being within-subject variables (see Table 1, Figure 3).

For RT, there were main effects of Block and of Transition. Responses were faster for the first and second go/nogo blocks (*M*s = 534 and 545 ms, respectively) than for the full-task block (*M* = 618 ms), and for repeat trials (*M* = 544 ms) than for switch trials (*M* = 587 ms), yielding TSCs. These results are consistent with Experiment 2. However, Transition interacted with Block: TSC was 11 ms for the first go/nogo block, which was not significant (*p* = .254), but it was 49 ms for the second go/nogo block and 71 ms for the full-task block, both of which were significant (*p*s < .002).

For PE, there were a main effect of Block, but not of Transition. Responses were most accurate for the second go/nogo task (*M* = 4.83%), intermediate for the first go/nogo block (*M* = 5.11%), and least accurate for the full-task block (*M* = 8.16%), as found in Experiment 2. Although the overall TSC was .70% for the first go/nogo block, −0.61% for the second go/nogo block, and 4.11% for the full-task block, the effect was significant only for the full-task block (*p* < .001).

For both RT and PE, the results differed from those of Experiment 2: In contrast to Experiment 2, TSC in Experiment 3 was not significant in the first go/nogo block for both RT and PE. This outcome is consistent with the possibility that, in Experiment 2, participants inferred and anticipated the second of the two tasks from the explicit task cues in the first block already, even though they were not yet instructed on it, whereas participants in Experiment 3 could not, and indeed did not. Taken together, these results imply that merely knowing the existence of two different tasks (assigned to two different task cues) can be sufficient to obtain TSCs even without a co-actor performing the second task. This finding has important implications for the interpretation of TSC in joint-task settings.

## General discussion

Two major variations of joint task switching have been used in previous studies that drew different conclusions about whether co-acting individuals shared the same task-sets. Our own previous studies tested a condition in which two actors performed the same set of two tasks, which provided no evidence supporting shared task-sets between co-actors (Yamaguchi et al., 2017b, 2019). Dudarev and Hassin (2016) and Liefooghe (2016) tested a condition in which two actors performed different tasks, and this procedure yielded costs of switching tasks in a joint-task setting but not in a solo-task setting. Dudarev and Hassin concluded that co-actors shared task-sets, although Liefooghe appeared more sceptical. If the former authors are correct and TSC in a joint task switching truly reflects shared task-sets between co-actors, the procedure would provide an important tool to investigate the nature of task sharing. Therefore, the present study aimed at replicating the findings from these two reports and further explored the source of TSC in joint task switching.

The main source of the discrepancies came from the observations of Dudarev and Hassin and of Liefooghe that TSCs were obtained in their joint condition but not in the solo condition. We thus attempted to replicate these outcomes in Experiment 1 by using a version of joint task switching similar to Dudarev and Hassin and Liefooghe’s procedures. However, in contrast to the previous two studies, we did not obtain any evidence suggesting that TSCs may be obtained in the joint, but not in the solo condition—if anything, we found that TSCs were larger in the solo condition. On the one hand, our findings thus replicate the observations of Dudarev and Hassin (2016) and Liefooghe (2016) that TSCs can be obtained under conditions in which the previous task was carried out by another person. On the other hand, however, this observation is not sufficient to argue that people perform the other person’s task or shared the task-sets, as speculated by Dudarev and Hassin. If actors would really mentally perform another person’s task under the conditions being tested here, they should exhibit much larger TSCs and should show such effects only in joint, but not in solo conditions.

Experiments 2 and 3 further examined why TSCs might occur in solo conditions. Experiment 2 used the same task cues as in Experiment 1, which denoted the two different tasks explicitly, and yielded TSC even before participants were instructed on the second of the two tasks. Experiment 3 used arbitrary symbols as task cues to conceal the two tasks entirely and yielded no TSC before participants were instructed on the second task. These results suggest that participants in Experiment 2 spontaneously inferred from the explicit task cues that they will be facing two different tasks assigned to the two task cues and actively prepared the task that they were not yet supposed to carry out, at least to some degree—a process that we successfully prevented in Experiment 3 by using arbitrary task cues that did not reveal the nature of the second task. This means that TSC can be obtained as long as participants know or infer that two task cues indicate two different tasks, even though they are not yet informed about the details of the second task.

Notwithstanding the possibility that what looks like TSCs may actually reflect more general switch-unrelated processes (see below), we can imagine at least two scenarios that may lead to such spontaneously generated switch costs. First, Cohen-Kdoshay and Meiran (2009) have argued that participants can quickly generate task representations that are fully operational even before the very first trial of a task. Along these lines, our participants might have generated rudimentary representations for the second task even before carrying out the first task. While the infrastructure of this task representation could not yet be complete, given that the corresponding stimulus–response mappings were not yet instructed, binding the cue to this rudimentary task-set might have been sufficient for the cue to activate the set, which then would conflict with the set for the first task—resulting in TSCs. Second, participants are likely to have carried out tasks involving critical stimulus colour or shape information before, which would imply that they brought stored task-sets with shape or colour as important ingredients or trigger conditions (Schumacher & Hazeltine, 2016; Waszak et al., 2003), so that even the not-yet-instructed task cue would trigger the retrieval of a previously acquired task-set that would now compete with the set of the first task—also resulting in TSCs.

Both scenarios would explain why Dudarev and Hassin did not find switch costs when two actors performed the same task because the task cues in such a condition were not associated with different tasks. At the same time, it is curious that the same authors failed to find any TSCs in their solo condition because their participants had received practice on both tasks prior to the first test block, regardless of whether they were assigned to the solo or joint condition. As Liefooghe suggested, some design features may have an impact on the degree to which a present co-actor attract attention to a degree that impairs the actor’s efficiency to retrieve task rules and to discriminate between the two actors in switch trials. For instance, the use of arbitrary task cues would make it easier to ignore the task that is not being performed in the given block, which may explain the lack of TSC in the solo condition of Dudarev and Hassin’s, who used arbitrary shapes as task cues. Also, switch trials have been found to render the cognitive system particularly vulnerable to the impact of irrelevant information (Waszak et al., 2003), so that the possible attention-distracting impact of a present co-actor may affect switch trials more than repeat trials (Dreisbach, 2012). If so, the differences we obtained between switch and repeat trials may not reflect true TSCs but, rather, the greater sensitivity of cognitive-control processes to irrelevant information in switch trials as compared to repeat trials.

Furthermore, as we pointed out earlier, it is important to keep in mind that there are at least two confounding factors in TSCs in the current procedure. First, task switching also required actor switching as two tasks were assigned to different actors. Second, task/actor switching was also confounded by the type of the preceding trial, as switch trials were trials that followed a nogo trial while repeat trials were trials that followed a go trial. Previous studies have indicated some evidence that RT tends to be longer after nogo trials than after go trials, regardless of task/actor switching (e.g., Nieuwenhuis et al., 2003; Verbruggen et al., 2016). What looks like TSCs in the present study may thus actually reflect general slowing after nogo trials or priming after go trials.

This explanation would also reconcile our findings with many previous studies using a go/nogo signal in a “solo” task switching procedure, in which TSCs were not obtained after a nogo trial in a “solo” task-switching procedure (e.g., Lenartowicz et al., 2011; Schuch & Koch, 2003; Verbruggen et al., 2005). The lack of TSCs after a nogo trial is often taken as evidence suggesting that TSCs are generated by response selection: True TSCs may still require previous response selection under another task-set, and thus may not occur after nogo trials. Therefore, there are good reasons to suspect that TSCs in the current version of joint task switching originated from processes other than those that generate true TSCs. In any case, it is clear that the present results provide little evidence for shared task-sets in joint task switching, and further investigations are needed to separate true from apparent TSC and to explore the possibility of the spontaneous creation and/or retrieval of task-sets.

## Notes

1. We also analysed the same data including Block Order (solo-task block first vs. joint-task block first) as a between-subject variable. The block order was determined randomly, and 24 pairs started with the solo-task block and 13 started with the joint-task block. The only interaction involving Block Order was that with Block, *F*(1,72) = 5.51, *MSE* = 2,246.15, *p* = .022, $\eta_{p}^{2}$ = .071, for RT and, *F*(1,72) = 5.51, *MSE* = 8.93, *p* < .001, $\eta_{p}^{2}$ = .258, for PE. This indicated that responses were slower and less accurate for the task block that was performed first than for the task block that was performed later. No other interactions were significant, suggesting that switch costs did not depend on the task order.
